# Optimization of Laser Repair Process for Agricultural Machinery Parts Based on Genetic Algorithm

**DOI:** 10.3390/ma18040775

**Published:** 2025-02-10

**Authors:** Qing Yi, Fei Feng

**Affiliations:** College of Engineering, China Agricultural University, Beijing 100083, China; 2022312260214@cau.edu.cn

**Keywords:** laser directional energy deposition, genetic algorithm, preferred algorithm, orthogonal experiment, process parameters

## Abstract

Laser-directed energy deposition technology (LDED), a method for repairing worn agricultural machinery parts, is valued for its flexibility, efficiency, and economy. To improve the comprehensive quality of the parts repair layer and reduce the processing energy consumption and time, it is necessary to explore the influence law of process parameters and multi-objective optimization experiments. We used L_9_ (3^3^) orthogonal experiments to evaluate the effects of laser power, scanning speed, and powder feed rate on repair quality. Variance analysis assessed factor level impacts and a multi-objective optimization model was constructed and optimized using a genetic algorithm (GA). Then, a preferred algorithm is proposed to optimize and obtain the optimal process level. The results show that the cladding efficiency increases at first and then decreases with the increase in laser power, decreases with the increase in scanning speed, and increases with the increase in powder feed rate. The dilution rate decreases at first and then increases with the increase in laser power, increases with the increase in scanning speed, and decreases with the increase in powder feed rate. In addition, it is also affected by the interaction between scanning speed and powder feed rate. Taking the maximum cladding efficiency and the minimum dilution rate as the optimization objectives, the verification test was carried out with the process parameters of laser power 1684.7370 W, scanning speed 3.0175 mm s^−1^, and powder feed rate 1.5901 r min^−1^. The error rates of cladding efficiency and dilution rate were 3.98% and 4.89%, respectively, which confirmed the method’s effectiveness. The research results can provide a reference for the repair of worn parts of agricultural machinery, which is not only cost-effective but saves time, as well. The free formability of the LDED process also allows it to add special functions to simple damaged castings and forging parts during the repair process to improve their performance.

## 1. Introduction

Agricultural machinery is vital in developing agricultural production in many developing countries [1,2]. Due to agricultural machinery’s heavy workload and complex working environment, its essential parts are easy to wear and fail, seriously affecting its working efficiency and service life [3]. At present, the repair and remanufacturing of agricultural machinery mainly use laser-directed energy deposition technology (LDED). Because of its advantages of high flexibility, high efficiency, and sound economy, it is often used as the preferred method for rapidly remanufacturing damaged parts. The process parameters of the LDED process will directly affect the geometric characteristics, phase distribution, and comprehensive performance of the repaired part and then play a decisive role in the repair quality [4]. Therefore, optimizing the LDED process parameters to prepare remanufacturing components with excellent performance is necessary.

At present, many scientists have carried out relevant research on the optimization of process parameters in the laser cladding process. Peng et al. [5] prepared Stellite6 single cobalt-based alloy cladding layer on a WCB low carbon steel substrate. The orthogonal experiment was designed, and the experimental results were analyzed by variance analysis and RSM. The influence order of process parameters on the morphology of the cladding layer was determined, and the prediction model of geometric characteristics of the cladding layer was constructed; Zhao et al. [6] used the response surface method to optimize the process parameters of furnace-assisted laser cladding to manufacture 60WC coatings on 45 steel substrates. The results show that by adjusting the two parameters of laser scanning speed and preheating temperature, a coating with high hardness and no cracks can be obtained; Shu et al. [7] established a fitting regression model of laser cladding process parameters and cladding coating quality and proposed an optimal coating parameter solution method based on the MOGWO algorithm. The optimized dilution rate of the molten coating increased by 17.8%; Li et al. [8] prepared Ni60PTA coating on 45 steel plates and found that laser power was the main factor affecting the dilution rate, and the scanning speed had the greatest influence on the aspect ratio and contact angle. The coating prepared by RSM optimization has good quality, no cracks, no deformation, and no pores; Ma et al. [9] studied the effect of laser melting process parameters on coating quality. The results show that the defocusing amount has the greatest influence on the dilution rate, and the scanning speed has the greatest influence on the residual stress. The coating with excellent performance is produced by using the optimal process parameters optimized by particle swarm optimization; Zhang et al. [10] proposed MOSMA-SVR-POLC to achieve laser melting target prediction and process parameter optimization, which can predict the dilution rate, powder utilization rate, and processing efficiency, and improve the performance of molten coating by optimizing process parameters; Gao et al. [11] used regression analysis to establish a mathematical model between process parameters and cladding layer morphology and then used this model to predict the morphology of the cladding layer under the new combination of process parameters.

This paper studies the optimization of process parameters of laser remanufacturing LDED technology. First, nine orthogonal experiments with three factors and three levels were designed. The laser power (LP), scanning speed (SS), and powder feed rate (V) were used as input variables, and the cladding efficiency (E) and dilution rate (DR) were used as response targets. The target model between LDED process parameters and repair surface performance indicators was established. Then, the process parameters are used as independent variables, and the GA genetic algorithm carries out the multi-objective optimization. Then, a preferred algorithm is proposed to optimize, and a multi-objective optimization model is established to find the optimal parameters and obtain the optimal process parameter combination.

## 2. Materials and Methods

### 2.1. Experimental Materials

Since the AISI 1045 steel has many applications in agricultural machinery production, it is selected as the matrix material in this experiment, and Inconel718 powder is chosen as the cladding powder. Inconel718 has excellent high-temperature performance, good wear resistance, and corrosion resistance, which can meet the needs of surface performance strengthening of AISI 1045 steel and produce good metallurgical bonding with the AISI 1045 steel matrix. The chemical composition of Inconel718 high-speed steel powder is shown in Table 1:

### 2.2. Experimental Methods

**Figure 1:**
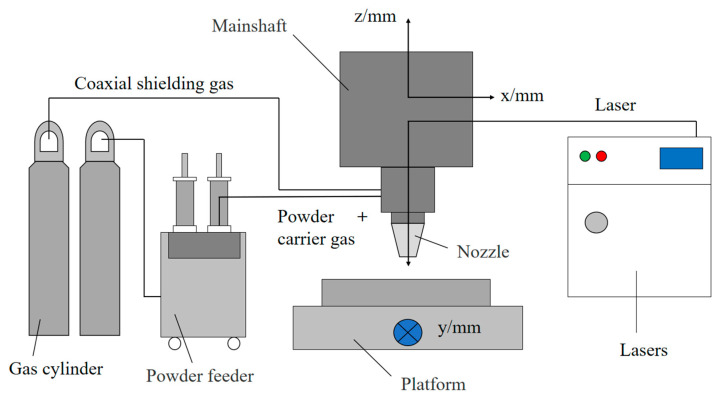
Schematic diagram of LDED repair device.

Figure 1 shows the schematic diagram of the LDED system. The laser cladding experimental equipment uses the LDM8060 high-power semiconductor fiber-coupled laser with a spot diameter of 3 mm. The powder feeding method uses the RC-PGF-D-2 of China Raycham (Nanjing Zhongke Yuchen) Company (Nanjing, China), and the double-bin negative pressure powder feeder is used for four-way coaxial powder feeding. The protective gas and powder feeding gas are nitrogen with a purity of 99.99%.

The preliminary test shows that the main factors affecting the quality of the repaired surface are laser power, scanning speed, and powder feed rate. Therefore, these three main process parameters are selected as the test factors. The laser spot adopts a circular spot of 3 mm, and the other process parameters are fixed. The levels of factors involved in the experiment are shown in Table 2.

In the field of remanufacturing laser cladding, the orthogonal-range analysis method is the most classical method in single-channel cladding, which has the advantages of improving test efficiency and reducing experimental costs. In this experimental design, the original 27 groups of experiments can be compressed into 9 groups. At the same time, the orthogonal experimental design is orthogonal so that the experiment has balanced dispersion and comprehensive comparability. This design method can produce a better experimental scheme with fewer experiments. The optimization parameters found by orthogonal experiments are consistent with the optimal conditions found by comprehensive experiments. The three-factor, three-level orthogonal test designed in this paper is as follows:

Before the experiment, the oil stain on the surface of the substrate was cleaned with anhydrous ethanol, and the Inconel718 powder was dried in a vacuum dryer at 120 °C for 120 min to remove the water vapor in the powder. The orthogonal test scheme of three factors and three levels was used for the experiment. The experimental samples were then subjected to wire cutting, mosaic, grinding, polishing, and other treatments to observe the macroscopic morphology. The three-dimensional microscopic system was used to measure the size of the cladding layer, and the mean value was measured several times. Table 3 shows the result of the experiment according to the orthogonal table L_9_ (3^3^) scheme.

### 2.3. Genetic Algorithm

The genetic algorithm performs selection, crossover, and mutation operations through population initialization and fitness function. Only individuals with good fitness values are retained for the next iteration until the final best individual is found [12]. Because it is always looking for the optimal value that satisfies the fitness function, this optimization method has good reliability and robustness. Compared with other optimization algorithms, the genetic algorithm has the following advantages:

1. The coding processing method and the fitness function optimization method are superior to other algorithms.
2. The application field is wide, and the nonlinear adaptability is strong.
3. The basic idea of the algorithm is simple, and parallel information processing is easy to use. In recent years, genetic algorithms have been applied to parameter optimization, control engineering, intelligent manufacturing, production scheduling, and other fields. The mathematical description when the genetic algorithm finds the maximum value of the function is:

(1) $$\begin{array}{l} \max\;f\left(X\right)\\ s.t.\;g\left(X\right)\leq 0\\ X\in R^{n} \end{array}$$

Formula (1) $X={\left[x_{1},x_{2},\cdot \cdot \cdot ,x_{n}\right]}^{T}$ represents the decision variable, *g*(*X*) represents the constraint condition, and *f*(*X*) represents the objective function. When solving the maximum value problem of the function, the fitness function represents the objective function, so that the fitness value reaches the maximum.

## 3. Results

### 3.1. Analysis of the Fitting Results of the Cladding Efficiency

**Figure 2:**
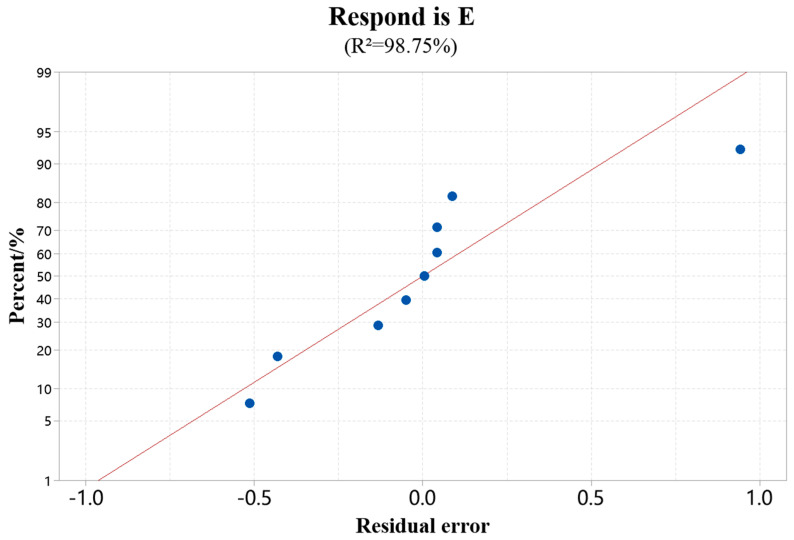
Standard probability diagram of cladding efficiency. (Data source: Table 3).

Figure 2 is the average probability diagram of the repair surface’s cladding efficiency. The residual distribution diagram shows that the data conforms to the normal distribution, which satisfies the dependence of the regression statistical analysis on the overall normality of the test results. The regression model of cladding efficiency established by stepwise regression analysis is shown in Formula (1), and the corresponding variance analysis is shown in Table 4.

(2) $$\begin{array}{l} E=-66.09440297+0.08868410\times LP-1.26370968\times SS\\ +6.94368131\times V-0.00002588\times LP\times LP \end{array}$$

From Table 4, in the single factor of the model, the *p* values of laser power, scanning speed, and powder feed rate are all less than 0.05, which significantly affects the cladding efficiency. The second-order *p* values of laser and laser power are less than 0.05, which also substantially affects the cladding efficiency. The regression model *p* is less than 0.05, and R^2^ is close to 1, indicating that the regression model of cladding efficiency has high fitting accuracy and recognition.

**Figure 3:**
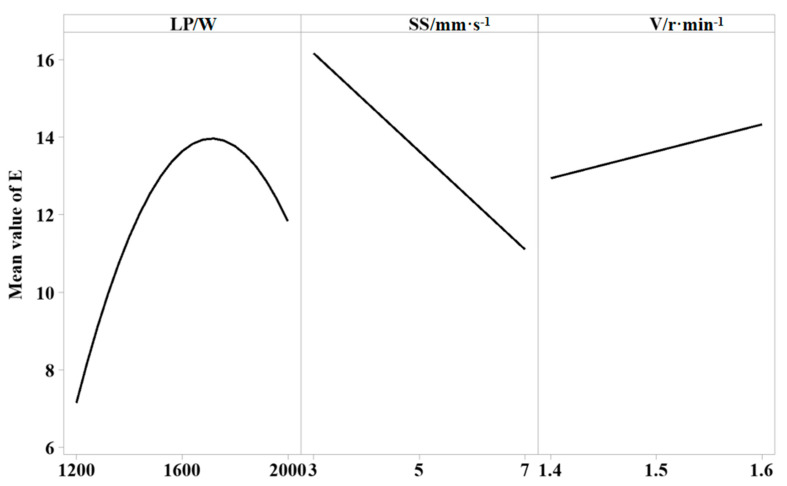
The central effect diagram of the mean value of cladding efficiency fitting.

Figure 3 is the central effect diagram of the mean value of the cladding efficiency of the repair surface. It can be seen that the cladding efficiency increases first and then decreases with the increase in laser power, decreases with the increase in scanning speed, and increases with the increase in powder feed rate. As the laser power increases, the energy absorbed by the material per unit area increases, the amount of powder melting increases, and the cladding efficiency increases with the increase in scanning speed because the increase in scanning speed leads to a decrease in the time of laser action in the molten pool, which leads to the incomplete melting of part of the powder in the outer contour of the cladding layer, the adhesion of the outer contour to the incomplete melting powder, and the cladding efficiency being reduced. With the increase in the powder feed rate, more powder is brought into the molten pool to melt, the area of the cladding layer increases, and the cladding efficiency also increases.

### 3.2. Analysis of Dilution Rate Model Fitting Results

**Figure 4:**
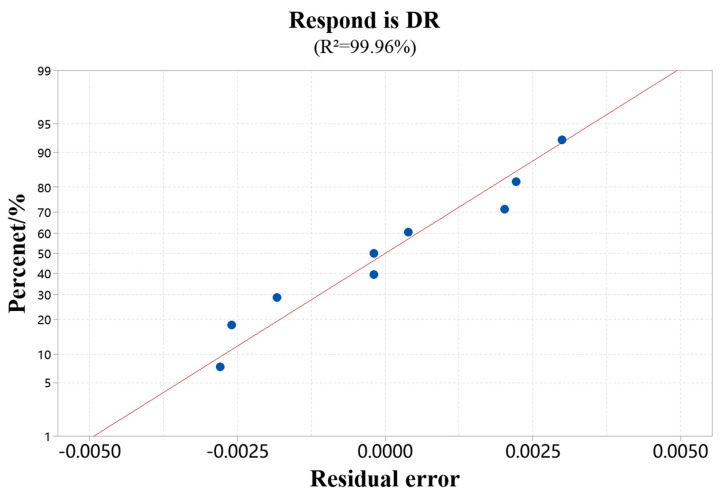
Dilution rate standard probability diagram. (Data source: Table 3).

Figure 4 shows the average probability diagram of the dilution rate. The residual distribution diagram shows that the data conforms to the normal distribution and satisfies the dependence of the regression statistical analysis on the overall normality of the test results. The regression model of the dilution rate established by stepwise regression analysis is shown in Formula (2), and the corresponding variance analysis is shown in Table 5.

(3) $$\begin{array}{l} DR=1.40444650-0.00095783\times LP-0.03444314\times SS-0.40298380\times V\\ +0.00000034\times LP\times LP+0.05856090\times SS\times V; \end{array}$$

**Figure 5:**
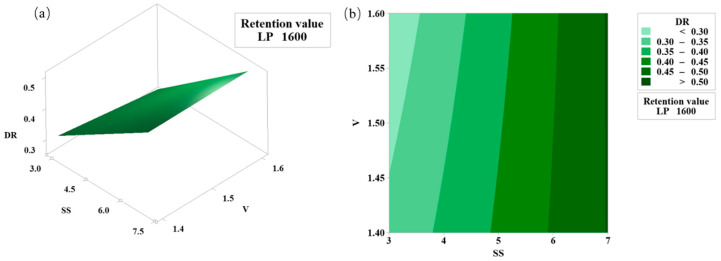
Dilution rate and SS, V surface figure and contour map (**a**) surface figure (**b**) contour map.

From Figure 5, in the single factor of the model, the *p* values of laser power and powder feed rate are less than 0.05, which has a significant effect on the dilution rate. Among the interaction factors, the interaction term of scanning speed and powder feed rate is substantial, and the second-order term *p* value of laser power and laser power is less than 0.05, significantly affecting the dilution rate. The regression model *p* is less than 0.05, and R^2^ is close to 1, indicating that the dilution rate regression model has high fitting accuracy and recognition.

Figure 5 shows the surface and contour diagrams between the dilution rate, scanning speed, and powder feed rate. The interaction between the scanning speed and the powder feed rate affects the dilution rate. It can be seen from Figure 5 that when the scanning speed is low, with the increase in the powder feed rate, the increase in the powder makes the cladding layer absorb more laser energy per unit area. The powder injected into the molten pool per unit area is reduced. The existence time of the molten pool is shorter, and the heat input is also reduced accordingly, resulting in a decrease in the dilution rate. The more considerable scanning speed will decrease the laser’s time acting on the molten pool and cause the powder to melt insufficiently. The powder accumulation phenomenon will occur by increasing the powder feed rate, decreasing the cladding layer area, and increasing the dilution rate [13].

**Figure 6:**
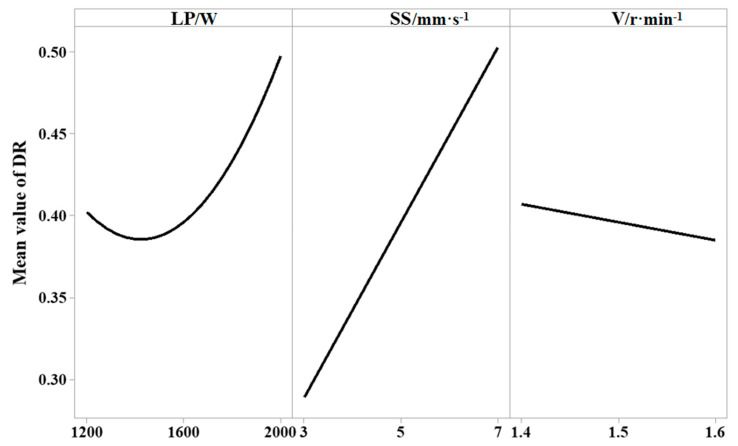
Dilution rate fitting mean central effect diagram.

Figure 6 shows the central effect diagram of the dilution rate fitting mean. It can be seen that the dilution rate decreases first and then increases with the increase in laser power, increases with the increase in scanning speed, and decreases with the increase in powder feed rate. At lower laser power, the heat input is lower, and the heat absorbed by the deposited powder and the substrate is also lower. Therefore, the depth of the molten pool becomes smaller, and the dilution rate decreases. With the increase in laser power, the heat gradually increases, resulting in a greater depth of the molten pool. At the same time, the amount of deposited powder that needs to be melted and mixed with the melted substrate increases, resulting in an upward trend in the dilution rate [14]. As the scanning speed increases, the laser energy is mainly absorbed by the substrate rather than the powder, so the dilution rate increases. The increase in powder feeding rate has a positive effect on the decrease in dilution rate. A higher powder feed rate will lead to more energy density absorbed into the powder, reducing melt penetration and decreasing the dilution rate [15].

## 4. Discussion

### 4.1. Multi-Objective Optimization

Based on the model obtained from the experiment, multi-objective optimization combined with actual production and life is an essential prerequisite for industrial application. At present, most of the multi-objective optimization can be expressed in the form of:

(4) $$\left\{\begin{array}{l} \min\left[f_{1}(x),f_{2}(x),f_{3}(x),\ldots ,f_{n}(x)\right]\\ lower\leq x\leq upper\\ A\times X\leq b\\ Aeq\times X=beq \end{array}\right.$$

where *x* is the decision vector, is the expression of the *f (x)* objective optimization function, *A* and *Aeq* are the constraint matrices of the optimization objective function, and the constraint matrix and the constraint domain jointly determine the feasible region of the decision vector. In the case of multi-objective optimization, there must be some contradictions in multi-objective function optimization. Finding the non-inferior (Pareto optimal solution) best meets the situation is the key to multi-objective optimization.

In additive manufacturing, the quality and performance of the repaired forming surface are coupled by various process parameters. Based on the repair process, the quality of the repaired surface can be reflected by its geometric characteristics. The depth of the repair surface affects the dilution rate, which reflects the bonding degree between the repair layer and the substrate. The lower dilution rate also means that the effect on the substrate is minor, which can reduce the energy waste caused by the poor combination of process parameters [16]. To improve production efficiency, cladding efficiency f1 was selected to maximize the optimization goal. The dilution rate *f*_2_ is used as an essential indicator to judge the weakening of the substrate during the repair process, and depreciation is the optimization goal. Therefore, the optimization model of this paper is shown in Formula (4):

(5) $$\left\{\begin{array}{l} \max[f_{1}(LP,SS,V)]\\ \min[f_{2}(LP,SS,V)] \end{array}\right.$$

#### 4.1.1. The GA Algorithm

The genetic algorithm is an optimization method suitable for global search, which is formed by simulating the process of biological heredity and evolution in nature. The genetic algorithm first determines the initial population and then produces an iteration through the replication, crossover, and mutation operations in the genetic algorithm. In continuous iterative evolution, the population will retain the individuals with better fitness, eliminate the individuals with poor fitness, and select the best individuals through multiple iterative evolutions. It can solve the optimization problem. Therefore, a genetic algorithm can be used to solve the problem of finding the optimal solution. The algorithm has a strong global search ability and is widely used in various fields. It can find the best solution in multi-objective optimization problems. Compared with the traditional algorithm, the genetic algorithm cannot easily fall into the local optimal solution, reliable and stable [17].

The Pareto Fraction specifies how many proportions of individuals should be retained into the next generation based on non-dominated sorting in each iteration. The smaller Pareto Fraction value means that only the best individuals will be retained, which can accelerate convergence but may lead to the loss of population diversity. The choice of 0.05 means that only 5% of individuals are considered non-dominated, which helps to maintain the competitiveness of the population; population size determines the number of individuals in each generation. Larger populations can provide more genetic diversity, which helps the algorithm to explore the solution space but also increases the computational cost. Two hundred is a moderate population size, which can provide sufficient diversity while maintaining computational efficiency. Generations determine the algebra of the algorithm. More algebras can give the algorithm more time to converge to the optimal solution, but it may also lead to overfitting and waste of computing resources. The 200th generation is a common choice, which is usually sufficient to converge the algorithm while avoiding unnecessary calculations. StallGenLimit means that the algorithm stops when no better solution is found in many generations. This parameter prevents the algorithm from continuing to run when it has converged, thereby saving computing resources. The 200-generation stagnation limit is a relatively loose setting that allows the algorithm to continue searching without improvement for a long time. TolFun is a convergence criterion used to determine when to stop the algorithm. If the change of the objective function value in successive iterations is less than this tolerance, the algorithm will stop. Here, 1 × 10^−5^ is a small tolerance, which means that the algorithm needs to converge very accurately, which helps to find high-quality solutions.

**Figure 7:**
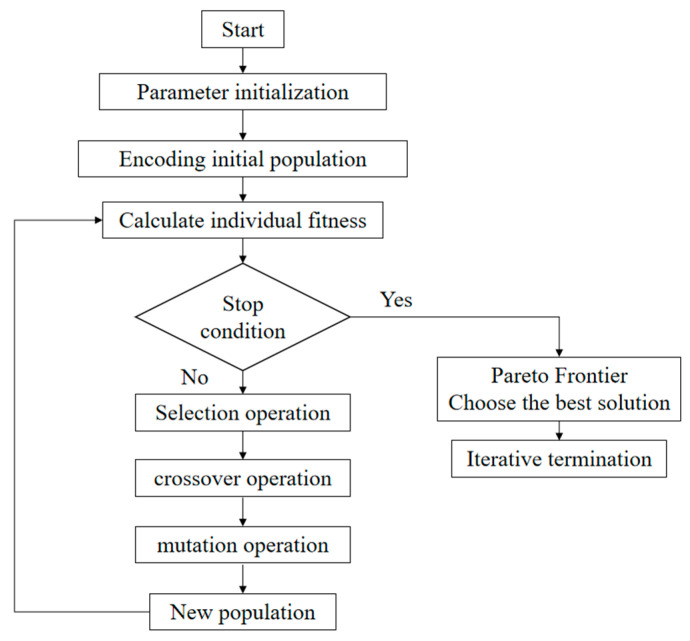
The GA algorithm flow chart.

**Figure 8:**
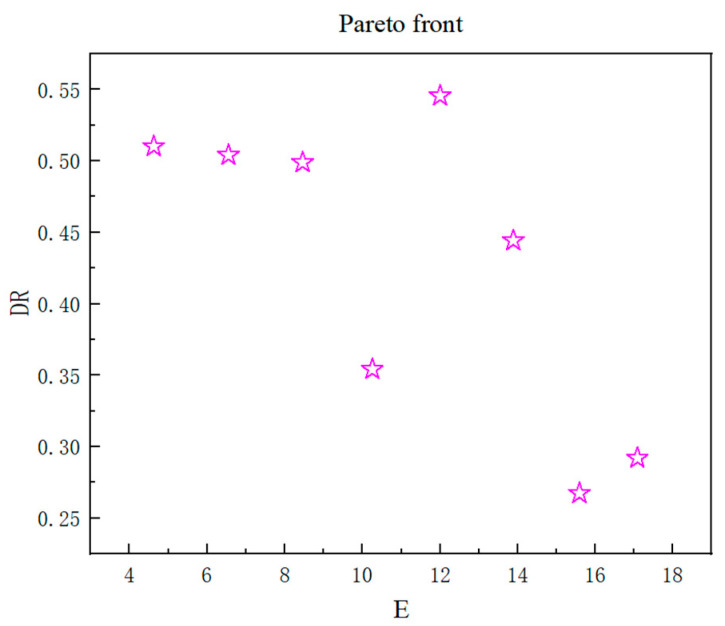
Pareto front of the GA algorithm.

The optimal individual coefficient (Pareto Fraction) in the GA algorithm is set to 0.05; the population size is 100; the maximum number of generations is 200. Taking the minimum value of the objective function as the objective, the Gamultiobj function is introduced, and the GA optimization algorithm is used to optimize the multi-objective optimization of the repair forming index. The algorithm flow chart is shown in Figure 7, the Pareto frontier of the optimization result is shown in Figure 8, and the Pareto frontier optimization result data are shown in Table 6.

#### 4.1.2. Optimal Selection Calculation Method

After the multi-objective optimization, a weighted scoring calculation method is proposed to optimize the target results. After the Pareto optimization solution of the target is obtained, the index is forwarded and standardized.

Suppose that there are *i* optimization objects and j optimization combinations. Firstly, the response values are transformed according to the characteristics of large and small. The processing method is shown in (5), where *x* and *x′* are the transformation results of large and small, respectively, and all the indices are transformed into vast and unified index types.

(6) $$\left\{\begin{array}{l} \mathrm{maximization}:x=a\\ \mathrm{minimization}:x^{\prime }=1-a \end{array}\right.$$

Standardization is performed column by column, data processing refers to the calculation method of the first column in Formula (7), and a matrix of *i* optimal combinations and j optimization indicators is constructed;

(7) $$z_{i1}=\frac{x_{i1}}{\sum_{n=1}^{i}x_{i1}}$$

(8) $$Z=\left(\begin{array}{ccc} z_{11} & \ldots & z_{1j}\\ \vdots & \ddots & \vdots \\ z_{i1} & \cdots & z_{ij} \end{array}\right)$$

The maximum value *Z*^+^ and the minimum value *Z*^−^ are defined. According to the calculation results of Equations (7) and (8), the distance *D*^+^ between each index and the maximum value and the distance *D*^−^ between each index and the minimum value are calculated, respectively, and the distance evaluation score *D_ij_* of each index is obtained.

(9) $$Z_{j}^{+}=\max\left\{z_{1j},z_{2j},\ldots ,z_{ij}\right\}$$

(10) $$Z_{j}^{-}=\min\left\{z_{1j},z_{2j},\ldots ,z_{ij}\right\}$$

(11) $$D_{ij}^{+}=\sqrt{{\left(Z_{j}^{+}-z_{ij}\right)}^{2}}$$

(12) $$D_{ij}^{-}=\sqrt{{\left(z_{ij}-Z_{j}^{+}\right)}^{2}}$$

(13) $$D_{ij}=\frac{D_{ij}^{-}}{D_{ij}^{+}+D_{ij}^{-}}$$

For each index, the calculation formula of information entropy *E_j_* is:

(14) $$E_{j}=-\frac{1}{\ln i}\sum_{n=1}^{i}s_{ij}\ln s_{ij}$$

(15) $$s_{ij}=\frac{z_{ij}}{\sum_{n=1}^{j}z_{ij}}$$

The weight *w_j_* is defined as

(16) $$w_{j}=\frac{1-E_{j}}{\sum_{n=1}^{j}\left(1-E_{j}\right)}$$

Finally, the score *S_i_* of each group is calculated and sorted one by one. The calculation results are shown in Table 7.

(17) $$S_{i}=\sum_{n=1}^{j}w_{j}D_{ij}$$

According to Table 7, it is not difficult to conclude that the optimal process parameter combination is laser power 1684.7370 W, scanning speed 3.0175 mms^−1^, powder feed rate 1.5901 r min^−1^, and the cladding efficiency is 17.0865 mm^3^ s^−1^. The dilution rate is 0.2921.

### 4.2. Experimental Validation

**Figure 9:**
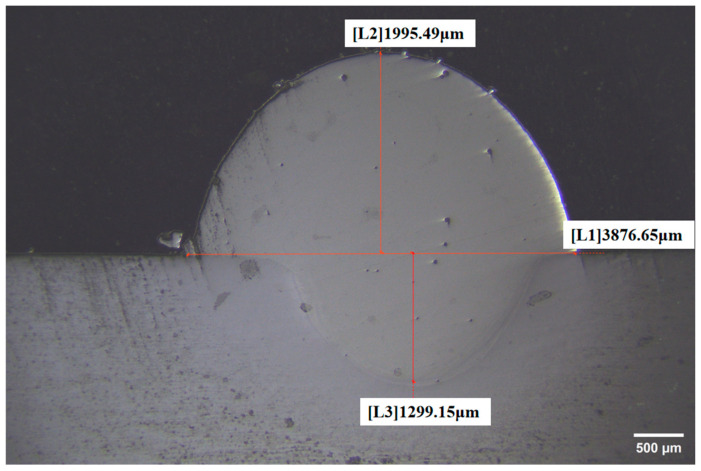
The cross-sectional topography of the verification group.

The process parameters needed to be experimentally verified to verify the rationality of the algorithm model of the cladding process parameters established by the cladding efficiency and dilution rate of the repaired surface of the material cladding. The highest score parameter combination is adopted. The selected process parameters are laser power 1684.7370 W, scanning speed 3.0175 mm s^−1^, and powder feed rate 1.5901 r min^−1^. The verification results are shown in Table 8. Through the calculation, the cladding efficiency and dilution rate errors are 3.98% and 4.89%, respectively. According to the error analysis, the established model has high prediction accuracy and can effectively realize the accurate prediction of cladding efficiency and dilution rate. The cross-sectional morphology of the sample is shown in Figure 9.

The macroscopic morphology of the cross-section of the optimized cladding layer was observed, as shown in Figure 9. The macroscopic morphology is a typical ‘crescent’ shape, and the cladding layer is closely combined with the substrate without cracks. Due to the limitation of experimental cost and experimental conditions, it is not possible to test under actual conditions, and further research will be carried out later. Referring to the existing related research [14,18], the hardness of the remanufacturing area and the tensile strength, tensile strength, and plasticity of the joints can be measured by systematically analyzing the microstructure and mechanical properties of the repaired area. By testing the mechanical properties of the remanufactured teeth and measuring the three-dimensional dimensions, it is further verified that it meets the maintenance requirements and significantly prolongs the service life of the repaired parts.

## 5. Conclusions

1. The laser remanufacturing process was used to successfully realize the forming of the repair surface, and the variance analysis was carried out based on the orthogonal test data to obtain the influence of the process parameters of the remanufacturing process on the single-channel repair characteristics. The analysis shows that with the increase in laser power, the energy absorbed by the material per unit area increases, the amount of powder melting increases, the cladding efficiency increases, the depth of the molten pool increases from decrease to increase, and the dilution decreases first and then increases. With the increase in scanning speed, the time of laser action in the molten pool decreases, the energy is mainly absorbed by the substrate rather than the powder, the cladding efficiency decreases, and the dilution rate increases. With the increase in the powder feed rate, more powder is brought into the molten pool to melt, the area of the cladding layer increases, the cladding efficiency increases, the melting penetration phenomenon decreases, and the dilution rate decreases.
2. The GA algorithm is used to carry out the multi-objective optimization, taking the maximum cladding efficiency and the minimum dilution rate as the optimization objectives. Then, a preferred algorithm is proposed. The optimal process parameters were laser power 1684.7370 W, scanning speed 3.0175 mm s^−1^, and powder feeding rate 1.5901 r min^−1^. According to the predicted and experimental values, the cladding efficiency and dilution rate error rates were 3.98% and 4.89%, respectively. Compared with the prediction group and the verification group, the optimization effect of the method on the performance of the repaired surface was confirmed. It provides reference significance for the repair of worn parts of agricultural machinery.

## Figures and Tables

**Table 1:** Chemical constituents of Inconel718 (wt%).

Fe	Cr	Ni	Nb	Mo	Al	Ti
Bal.	19.54	52.05	5.02	3.07	0.46	0.91

**Table 2:** Design of LDED orthogonal process parameters.

Factors	Levels		
	1	2	3
LP/W	1200	1600	2000
SS/mm·s^−1^	3	5	7
V/g·min^−1^	1.4	1.5	1.6

**Table 3:** Orthogonal test results.

Sample	LP/W	SS/mm·s^−1^	V/r·min^−1^	E/mm^3^·s^−1^	DR
1	1200	3	1.4	8.4694	0.3156
2	1200	5	1.5	8.0938	0.4027
3	1200	7	1.6	4.8864	0.5120
4	1600	3	1.5	16.1110	0.2923
5	1600	5	1.6	14.3328	0.3823
6	1600	7	1.4	10.4547	0.5020
7	2000	3	1.6	15.0983	0.3680
8	2000	5	1.4	11.2272	0.5107
9	2000	7	1.5	9.1731	0.6027

**Table 4:** Analysis of variance of cladding efficiency.

Source	Freedom	Adj SS	Adj MS	F Value	*p* Value
Regression	4	108.42022	27.10506	79.04082	0.00046
LP	1	39.12059	39.12059	114.07922	0.00044
SS	1	38.32709	38.32709	111.76530	0.00045
V	1	2.89288	2.89288	8.43591	0.04392
LP × LP	1	34.30432	34.30432	100.03453	0.00056
Error	4	1.37170	0.34292		
Total	8	109.79192			
R^2^	98.75%				

**Table 5:** DR variance analysis of dilution rate.

Source	Freedom	Adj SS	Adj MS	F Value	*p* Value
Regression	5	0.087080	0.017416	1447.316556	0.000029
LP	1	0.003471	0.003471	288.441483	0.000445
SS	1	0.000042	0.000042	3.500128	0.158118
V	1	0.000493	0.000493	40.998990	0.007717
LP × LP	1	0.004641	0.004641	385.659449	0.000288
SS × V	1	0.000274	0.000274	22.799184	0.017456
Error	3	0.000036	0.000012		
Total	8	0.087116			
R^2^	99.96%				

**Table 6:** The GA algorithm optimization data table.

Sample	LP/W	SS/mm·s^−1^	V/r·min^−1^	E/mm^3^·s^−1^	DR
1	1219.5413	6.9873	1.4236	4.6239	0.5102
2	1776.1387	6.9973	1.5937	12.0020	0.5456
3	1684.7370	3.0175	1.5901	17.0865	0.2921
4	1698.4625	5.5685	1.5914	13.8874	0.4443
5	1272.8394	4.3645	1.5716	10.2544	0.3542
6	1260.0178	6.9475	1.5506	6.5482	0.5041
7	1219.5413	6.9873	1.4236	4.6239	0.5102
8	1381.2369	6.9934	1.4793	8.4593	0.4989
9	1776.1387	6.9973	1.5937	12.0020	0.5456
10	1471.1223	3.0164	1.5902	15.5914	0.2674

**Table 7:** Weighted scores and ranking of cladding efficiency and dilution rate.

Sample	E/mm^3^·s^−1^	DR	Score	Rank
1	4.6239	0.5102	0.0497	9
2	12.0020	0.5456	0.3609	5
3	17.0865	0.2921	0.9653	1
4	13.8874	0.4443	0.5953	3
5	10.2544	0.3542	0.5440	4
6	6.5482	0.5041	0.1524	8
7	4.6239	0.5102	0.0497	9
8	8.4593	0.4989	0.2531	7
9	12.0020	0.5456	0.3609	5
10	15.5914	0.2674	0.9269	2

**Table 8:** Comparison of prediction results optimization and experimental verification results.

Comparison	LP/W	SS/mm·s^−1^	V/r·min^−1^	E/mm^3^·s^−1^	DR
Prediction	1684.7370	3.0175	1.5901	17.0865	0.2921
Validation	1684.7370	3.0175	1.5901	17.7948	0.3071

## Data Availability

The original contributions presented in this study are included in the article, further inquiries can be directed to the corresponding author.
